# Overview on the Bacterial Iron-Riboflavin Metabolic Axis

**DOI:** 10.3389/fmicb.2018.01478

**Published:** 2018-07-05

**Authors:** Ignacio Sepúlveda Cisternas, Juan C. Salazar, Víctor A. García-Angulo

**Affiliations:** Programa de Microbiología y Micología, Instituto de Ciencias Biomédicas, Universidad de Chile, Santiago, Chile

**Keywords:** riboflavin, iron, uptake, redox, cofactor

## Abstract

Redox reactions are ubiquitous in biological processes. Enzymes involved in redox metabolism often use cofactors in order to facilitate electron-transfer reactions. Common redox cofactors include micronutrients such as vitamins and metals. By far, while iron is the main metal cofactor, riboflavin is the most important organic cofactor. Notably, the metabolism of iron and riboflavin seem to be intrinsically related across life kingdoms. In bacteria, iron availability influences expression of riboflavin biosynthetic genes. There is documented evidence for riboflavin involvement in surpassing iron-restrictive conditions in some species. This is probably achieved through increase in iron bioavailability by reduction of extracellular iron, improvement of iron uptake pathways and boosting hemolytic activity. In some cases, riboflavin may also work as replacement of iron as enzyme cofactor. In addition, riboflavin is involved in dissimilatory iron reduction during extracellular respiration by some species. The main direct metabolic relationships between riboflavin and iron in bacterial physiology are reviewed here.

## Introduction

Redox reactions involving electron transfers among molecules are highly required in central biological processes, from CO_2_ fixation and oxidative phosphorylation to protein folding and cell signaling. Many of the enzymes that catalyze biological electron transfer utilize diverse vitamins and/or metals as cofactors. Riboflavin and iron are the main cofactors, each one assisting about 17% of the cofactor-requiring enzymes (Monteverde et al., 2017). Within a cell, iron can function in a number of different forms, such as being part of heme, forming iron-sulfur clusters or as mono- and di-nuclear non-heme iron in some proteins (Caza and Kronstad, 2013). Riboflavin, also known as vitamin B2, is usually transformed into riboflavin-5′-phosphate (FMN) and flavin adenine dinucleotide (FAD), which are the main flavin cofactors.

Iron is mainly present in two oxidation states, ferrous (Fe^2+^) and ferric (Fe^3+^). The interconversion between these two states enables single electron transfers (Sheldon et al., 2016). With a few exceptions, iron is essential for bacteria. Although iron is one of the most abundant elements on earth, due to the oxidative atmosphere and basic pH the insoluble Fe^3+^ form is predominant, mainly forming iron oxides. Moreover, under physiological conditions, the iron concentration is some orders of magnitude lower than that needed to support bacterial growth (Sheldon et al., 2016). Thus, iron bioavailability is usually limiting. Hence, bacteria have evolved an assortment of strategies to solubilize and internalize iron and to compete for iron against a host or other microorganisms. These include the expression of siderophores, which are low molecular mass, iron-chelating molecules. Siderophores have a high affinity for ferric ion and can internalize environmental iron or hijack Fe^3+^ bound to mammalian host proteins such as transferrin. Bacteria may also obtain iron through direct interaction of bacterial receptors with host transferrin, heme and hemoproteins and subsequent internalization and dissociation of their coordinated iron molecules. Many species can also produce iron reductases, which may be secreted or associated with the membrane, in order to reduce Fe^3+^ into its more soluble Fe^2+^ form and uptake it through ferrous ion transport systems (Sheldon et al., 2016; Butt and Thomas, 2017; Pi and Helmann, 2017a; Pokorzynski et al., 2017; Sánchez et al., 2017).

A riboflavin molecule consists of an isoalloxazine ring with a substitution at N10 with a ribityl chain. The main biological active forms of riboflavin are its phosphorylated derivative FMN and the adenylated derivative of this, FAD. Flavins may alternate between three redox forms: oxidized, one-electron reduced or two-electron reduced. This allows them to mediate both one-electron and two-electron transfer reactions as well as electron bifurcation processes, which consists in the branching of two electrons from a single donor into two different single-electron acceptors (Abbas and Sibirny, 2011; Haase et al., 2014; Beztsinna et al., 2016; Buckel and Thauer, 2018). Most bacteria synthesize riboflavin through the riboflavin biosynthetic pathway (RBP), which starts from guanosine-5′-triphosphate and ribulose-5-phosphate. The RBP has been extensively reviewed in previous works (Fischer and Bacher, 2005, 2011; Haase et al., 2014). The bacterial enzymes forming the RBP are encoded by the *ribABDEH* genes and may be localized within a single operon or scattered in the genome (Vitreschak et al., 2002; García-Angulo, 2017). Also, transport systems from different families may internalize riboflavin in bacteria (Vitreschak et al., 2002; Vogl et al., 2007; Rodionov et al., 2009; Hemberger et al., 2011; Deka et al., 2013; García Angulo et al., 2013; Sun et al., 2013; Gutiérrez-Preciado et al., 2015; Jaehme and Slotboom, 2015; Rodionova et al., 2015; García-Angulo, 2017). Thus, bacteria may obtain the riboflavin by *de novo* biosynthesis and/or by internalization from the environment.

Both iron and riboflavin may have been present in early earth and be recruited by primordial biological systems to perform biotic functions early in evolution. In the highly reductive atmosphere of the Archean, when life began, the abundant Fe^2+^ could have been the main electron donor for photosynthesis. Both cofactors participate in ancient biochemical pathways such as CO_2_ fixation, where the electron bifurcation capacity of flavins is required. (Sousa et al., 2013, 2018; Knoll et al., 2016; Monteverde et al., 2017; Sánchez et al., 2017). Thus, organisms across kingdoms have conserved a dependence on riboflavin and iron to perform basic processes, mainly based on their redox properties. Moreover, it has been documented a metabolic crosstalk between riboflavin and iron in a number of organisms including animals, plants, yeast, and bacteria (Welkie and Miller, 1960; Buzina et al., 1979; Charoenlarp et al., 1980; Powers et al., 1988; Crossley et al., 2007; Chazarreta-Cifre et al., 2011; Hsu et al., 2011; Zhang Y. et al., 2015; Chen et al., 2017; Xin et al., 2017). In many cases, these two cofactors are involved in similar processes, sometimes with enzymes requiring both cofactors at the same time. It has also been documented the replacement of enzymes on the basis of cofactor availability and the development of an iron-riboflavin regulatory feedback. This review focuses on current research on the main metabolic interrelationships between iron and riboflavin in bacteria.

## Iron-Riboflavin Regulatory Interplay in Bacteria

In bacteria, Fur is the main regulator of iron homeostasis. Fur is a transcriptional regulator that upon interaction with iron, binds to consensus DNA operators named Fur boxes close to promoters of target genes to regulate transcription. Bacterial Fur regulons include many iron uptake genes. Under low iron, iron dissociates from Fur leaving it unable to interact with Fur boxes and thus allowing the expression of such iron acquisition genes. When intracellular iron levels are sufficient, Fur binds iron and represses the genes coding for transport of iron, avoiding toxicity caused by a potential surplus. Excess iron is detrimental because of generation of reactive oxygen species through the Fenton reaction. Indirectly, Fur-iron may also positively regulate the expression of iron-storage and iron-utilizing proteins as well as iron efflux systems (Massé et al., 2007; Pi et al., 2016; Pi and Helmann, 2017a). Although initially recognized as a repressor, the Fur-iron complex is also able to directly activate genes not related to iron metabolism (Craig et al., 2011). Moreover, evidence for iron-independent Fur regulation has been obtained in some bacteria (Carpenter et al., 2009; Agriesti et al., 2014).

In many cases, the expression of RBP and riboflavin uptake genes are under the control of the FMN riboswitch (Vitreschak et al., 2002; Pedrolli et al., 2012; García Angulo et al., 2013; Pedrolli and Mack, 2014; Gutiérrez-Preciado et al., 2015; Pedrolli D.B. et al., 2015; Pedrolli D. et al., 2015; García-Angulo, 2017). This is a sequence conserved in the leader regions of messenger RNA which switches between alternative secondary structures depending on FMN binding status. Under high flavins concentrations, FMN binds the aptamer sequence within the riboswitch, promoting the formation of a secondary structure that blocks transcription, translation or both, of downstream coding sequences. When intracellular flavins concentration drop, FMN dissociates from the riboswitch, which allows genetic expression. This way, the expression of both riboflavin supply pathways, biosynthesis and uptake, is keenly linked to the intracellular flavin requirements. Nonetheless, not all bacterial RBP and riboflavin transport genes conserve a FMN riboswitch and likely, other species-specific regulatory traits may exist (García-Angulo, 2017).

It has long been known that iron physiological status influences riboflavin biosynthesis. A study on a *Clostridium acetobutylicum* strain used for large scale production of riboflavin, showed that the presence of iron decreased riboflavin yield, while the iron chelator bipyridine highly increased the production of the vitamin (Hickey, 1945). More recently, it was shown in this species that both iron starvation and Fur deletion highly increase the transcription of the RBP operon (Vasileva et al., 2012). Iron starvation induces the secretion of riboflavin in *Methylocystis* sp., a methanotrophic bacterium (Balasubramanian et al., 2010). Also, both iron deprivement and elimination of Fur increase the riboflavin uptake activity in *Campylobacter jejuni* (Crossley et al., 2007). Riboflavin production and transcription of the bifunctional *ribBA* gene are highly increased in low iron in *Helicobacter pylori*. In this species, a putative Fur box is found in *ribBA* (Worst et al., 1998). Transcriptomics approaches have also confirmed that iron and Fur negatively regulate the expression of the RBP operon in *C. acetobutylicum* and *Caulobacter crescentus* (da Silva Neto et al., 2013; Nguyen et al., 2016). In the latter, a putative Fur box is upstream of the RBP operon. In *C. acetobutylicum*, iron uptake genes and RBP genes are co-regulated by the carbon starvation regulator CsrA (Tan et al., 2015). These studies suggest that in some bacteria, a fall in iron supply may induce an increase in riboflavin biosynthesis and/or uptake. Nonetheless, iron effects over the expression of riboflavin provision genes are not always equal. *Vibrio vulnificus* is a bacterium causing wound infections and septicemia whose pathogenic potential is tightly related to the iron availability. In this pathogen, iron restriction was found to upregulate *ribH*, but to downregulate *ribE*, *ribA*, and *ribB* homologs, while elimination of Fur downregulated *ribA* and *ribB* but overexpressed *ribE* and *ribH* (Pajuelo et al., 2016). In *V. cholerae*, a phylogenetically related pathogen, the riboflavin regulon presents a high degree of overlap with the iron regulon (Mey et al., 2005; Sepúlveda-Cisternas et al., 2018). In this bacterium, iron regulates the expression of the RBP genes and of the *ribN* riboflavin importer in a riboflavin-dependent way. This regulation is gene-specific, as while iron repressed *ribB* in the presence of extracellular riboflavin, it induced the expression of *ribD* and *ribN* in the absence of exogenous riboflavin. Reciprocally, riboflavin repressed the expression of the *tonB1* gene encoding a protein involved in the function of various iron uptake systems, but only under iron-replete conditions (Sepúlveda-Cisternas et al., 2018). Thus, iron levels may regulate the status of riboflavin provision in a gene-specific fashion and reciprocally, riboflavin exerts regulatory effects over iron acquisition genes.

## Riboflavin Production as a Mean to Increase Iron Availability

**FIGURE 1:**
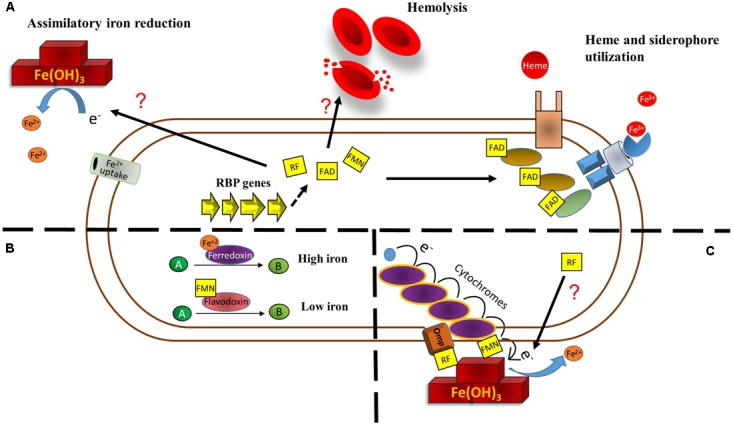
Main direct metabolic relationships among riboflavin and iron in bacteria. **(A)** Riboflavin may help overcome iron restriction status in three principal ways. Riboflavin increases extracellular iron reduction by a yet uncharacterized mechanism. This increases iron solubility and could make it prone to uptake by ferrous iron uptake systems. Increases in bacterial riboflavin expression produce a higher degree of hemolysis, which can increase hemoglobin availability. Also, flavins function as cofactor for enzymes involved in heme utilization and iron release from siderophores. **(B)** Under iron restriction, enzymes using iron as redox cofactor to catalyze a reaction may be replaced by enzymes that use flavins as cofactor. **(C)** Riboflavin increases dissimilatory ferric reduction. In *S. oneidensis*, extracellular ferric iron is used as final electron acceptor to complete the electron transport chain from quinol. This is achieved by a set of cytochromes and an outer membrane protein with the aid of flavins. In other bacteria, the mechanism has not been characterized.

Increases in riboflavin biosynthesis may help overcome iron restrictive conditions. *H. pylori ribBA* bifunctional gene has been shown to restore growth in low iron to a siderophore-deficient *Escherichia coli* strain. The mechanism through which riboflavin biosynthesis relieves iron stress in this species has not been fully elucidated. It was shown that the riboflavin production mediated by *H. pylori* RibBA increases extracellular iron reduction (Worst et al., 1998). Conversion of exogenous Fe^3+^ into Fe^2+^ increases iron solubility and may make it suitable for uptake through ferrous ion internalization systems. In *C. jejuni*, riboflavin biosynthesis also increases reduction of extracellular Fe^3+^ through an unknown mechanism (Crossley et al., 2007) (**Figure 1A**). In addition, both the bifunctional RibBA and a monofunctional RibA protein of *H. pylori* are able to confer hemolysis properties to *E. coli.* (Bereswill et al., 1998; Worst et al., 1998). Likely, hemolysis itself may be a strategy to increase iron bioavailability by releasing the iron contained in erythrocytes in an infection setting. Nonetheless, the mechanism through which riboflavin biosynthesis boosts hemolysis is not clear. Riboflavin has been shown to direct light-mediated rat and human erythrocytes hemolysis but only in the presence of other factors such as serum, oxygen, copper, azide, and aminophylline (Suzuki et al., 1982; Ali and Naseem, 2002; Ali et al., 2005). Moreover, riboflavin alone does not directly promote hemolysis in agar plates were *H. pylori* experiments were conducted (Bereswill et al., 1998). Thus, hemolysis may be given by augmented riboflavin biosynthesis intermediaries or increases in the expression and/or activity of a hemolysis factor (**Figure 1A**).

Riboflavin is also required as cofactor for enzymes involved in iron acquisition. In *Staphylococcus aureus*, FAD functions as cofactor for IruO, a reductase of IsdG and IsdI, which are enzymes required for the degradation of heme. IruO is also directly required for iron reduction during its release from siderophores (Kobylarz et al., 2017). In addition, two more other bacterial reductases required for iron release from siderophores depend upon FAD as cofactor, YqjH in *E. coli* (Bamford et al., 2008) and FscN in *Thermobifida fusca* (Li et al., 2015). Moreover, dihydroflavin produced by the NADH:flavin oxidoreductase FerA acts as the electron donor during reductive release of Fe^3+^ bound to siderophore in *Paracoccus denitrificans*. This process is necessary for this species to grow under iron limitation (Mazoch et al., 2004; Sedlácek et al., 2009; Sedláček et al., 2016). Thus, by increasing riboflavin production, bacteria may increase the activity of some components of iron acquisition pathways (**Figure 1A**).

## Riboflavin to Replace Iron as Cofactor

In many bacteria, iron restriction highly induces the expression of iron uptake systems and also triggers a response known as iron-sparing. This consists in the downregulation of non-essential iron-dependent enzymes in order to prioritize iron for fundamental functions. For example, iron-starving *Mycobacterium tuberculosis* and *Bacillus subtilis* downregulate aconitase and succinate dehydrogenase (Janagama et al., 2010; Smaldone et al., 2012), while *Agrobacterium tumefaciens* downregulates a system encoding a multicopper oxidase, cytochrome c_556_ and a NAD-dependent formate dehydrogenase complex, all of them needing iron as cofactor (Heindl et al., 2015). Strikingly, the iron-sparing response may also include the replacement of iron-cofactored enzymes by flavin-dependent enzymes. Early work showed that ferredoxin and flavodoxin alternate to catalyze a dehydration reaction depending on the iron status during fermentation of glutamate in *Acidaminococcus fermentans* (Thamer et al., 2003). In *C. acetobutylicum*, the expression of ferredoxin is decreased and a flavodoxin encoded by *fdl1* is highly induced under iron deficiency (Vasileva et al., 2012; Nguyen et al., 2016). The *fdl1* gene conserves a Fur box. In these conditions the riboflavin biosynthetic operon is also induced. In *B. subtilis*, the Fur-regulated flavodoxins YkuNOP are expressed under iron deprivement also likely to replace ferredoxin (Baichoo et al., 2002). Notably, in this species the YkuN and YkuP flavodoxins, which use FMN as prosthetic group, have been experimentally shown to substitute ferredoxin to transfer electrons for desaturation of membrane phospholipids (Chazarreta-Cifre et al., 2011) (**Figure 1B**). A recent study demonstrated that the *B. subtilis* Fur regulon is induced in three sequential steps, the first one including the expression of iron-uptake systems, while expression of flavodoxins putatively to replace ferredoxin is included in the second wave. The third stage is comprised by the downregulation of superfluous iron-dependent enzymes (Pi and Helmann, 2017b).

## Role of Riboflavin in Dissimilatory Iron Reduction

Under anoxic conditions, some bacteria are able to reduce extracellular insoluble metals as final electron acceptors to complete the respiratory chain. In this case, riboflavin may also work as intermediary for dissimilatory iron reduction and this phenomenon has been extensively studied in *Shewanella oneidensis*, a facultative anaerobe model for electrochemically active microbes (Kouzuma et al., 2018). *S. oneidensis* is able to reduce environmental Fe^3+^ and other metal oxides due to a system to transport electrons from the inner membrane across the periplasm and outer membrane to the metal surface. This pathway, known as the metal reducing (Mtr) pathway, consists of the multiheme domain c type cytrochromes CymA, MtrA, MtrC, and OmcA and the outer membrane porin MtrB. The inner-membrane CymA is proposed to transfer electrons from quinol to MtrA, which is localized in the outer membrane and interacting with MtrB. The MtrAB complex conveys the electrons to the MtrC and OmcA c cytrochromes in the bacterial surface which in turn act as reductases to transfer electrons to oxides (Brutinel and Gralnick, 2012; Shi et al., 2012; Breuer et al., 2015). Although MtrC and OmcA are able to directly transfer electrons to Fe^3+^ oxides, secreted riboflavin and FMN highly enhance this activity (Marsili et al., 2008; von Canstein et al., 2008). In this species, flavins are secreted through the Bfe exporter and flavin-mediated reduction accounts for most (up to 75%) of extracellular electron transfer to insoluble acceptors (Kotloski and Gralnick, 2013). Although originally a mechanism where freely diffusing flavins working as electron shuttles for Fe^3+^ reduction was proposed, later research documented that flavins directly interact with MtrC and OmcA to transfer electrons (Okamoto et al., 2013, 2014; Hong and Pachter, 2016; Babanova et al., 2017). While riboflavin binds OmcA (Hong and Pachter, 2016), FMN binds MtrC to form a highly reductive flavocytochrome (Edwards et al., 2015) (**Figure 1C**). The presence of Fe^3+^ in the medium increases the secretion of riboflavin and FMN (Wu et al., 2013).

Extracellular riboflavin as intermediary for dissimilatory iron reduction has been reported in other bacteria species. *Geothrix fermentans*, an isolate of an aquifer contaminated with petroleum, uses secreted riboflavin to shuttle electrons toward environmental Fe^3+^ (Mehta-Kolte and Bond, 2012). Carbohydrate oxidation is increased when using riboflavin as electron transfer mediator in the presence of crystalline Fe(OH)_3_ as extracellular electron sink in *Clostridium beijerinckii* and an uncharacterized novel rhizobial solventogenic bacterium (Popovic et al., 2017). Also, riboflavin is likely involved in the reduction of extracellular Fe^3+^-citrate and solid phase hydrous ferric oxide by *Desulfotomaculum reducens*, a sulfate-reducing Gram-positive species (Dalla Vecchia et al., 2014) and in anaerobic Fe^3+^ reduction by an alkaliphilic bacterial consortium (Fuller et al., 2014). Nonetheless, the mechanism through which these species achieve extracellular iron reduction with the aid of flavins has not been investigated (**Figure 1C**). Notably, flavins may also mediate electron transfer in the opposite direction. Riboflavin and FAD increase extracellular iron oxidation of stainless steel, a process that provides the electrons required for intracellular sulfur reduction in *Desulfovibrio vulgaris* (Zhang P. et al., 2015).

## Concluding Remarks

Many bacteria have developed a regulatory interplay on which iron levels influence the expression of riboflavin biosynthesis and uptake systems. This is probably the reflex of the fact that there is a number of physiological traits on which riboflavin and iron metabolism directly relate. First, riboflavin could help surpass eventual iron restriction conditions. Many species have complex life cycles, being able to colonize different niches with fluctuating physical conditions and nutrient availability. Thus, iron availability may change drastically. Riboflavin increases iron bioavailability and improves iron acquisition pathways. The ability of riboflavin to transfer electrons is used by some bacteria to reduce extracellular ferric oxides making it more suitable for uptake. Flavins are used as cofactor for some proteins directly involved in iron uptake, so increasing riboflavin concentration may also make the process more active. Also, some species may economize iron by replacing iron-requiring enzymes with enzymes that perform similar functions but use riboflavin as cofactor instead. This comprises a remarkable outline of the common redox properties of both molecules. Second, riboflavin participates in dissimilatory iron reduction. Riboflavin-mediated extracellular iron reduction is employed by some species to accomplish the respiratory electron chain. Altogether, this high degree of metabolic cross-talk may be reminiscent of an early recruitment of both molecules to perform related functions during biological redox reactions.

## Author Contributions

IS contributed to the general draft and designed the **Figure 1**. JS wrote the part corresponding to Fur regulation. VG-A wrote the main text. All authors discussed the whole review and agreed with the final version of the manuscript.

## Conflict of Interest Statement

The authors declare that the research was conducted in the absence of any commercial or financial relationships that could be construed as a potential conflict of interest.
